# Study on Environmental and Lifestyle Factors for the North–South Differential of Cardiovascular Disease in China

**DOI:** 10.3389/fpubh.2021.615152

**Published:** 2021-07-16

**Authors:** Mengqi Wang, Yi Huang, Yanxin Song, Jianwei Chen, Xiaoxiao Liu

**Affiliations:** School of Geographic Sciences, Nantong University, Nantong, China

**Keywords:** mortality rate, Northern China, Southern China, environmental factors, life style factors, cardiovascular disease

## Abstract

Human death and life span are closely related to the geographical environment and regional lifestyle. These factors considerably vary among counties and regions, leading to the geographical disparity of disease. Quantitative studies on this phenomenon are insufficient. Cerebrovascular and heart diseases are the leading causes of death. The mortality rate of cerebrovascular and heart diseases is statistically higher in northern China than in southern China; the *p*-value of *t*-test for cerebrovascular and heart diseases was 0.047 and 0.000, respectively. The population attribution fraction of 12 major risk factors for cardiovascular disease (CVD) in each province was calculated based on their exposure and relative risk. The results found that residents in northern China consume high sodium-containing food, fewer vegetables, and less sea food products, and tend to be overweight. Fine particulate matter is higher in northern China than in southern China. Cold temperatures also cause a greater number of deaths than hot temperatures. All these factors have resulted in a higher CVD mortality rate in northern China. The attributive differential for sodium, vegetable, fruit, smoking, PM_2.5_, omega-3, obesity, low temperature, and high temperature of heart disease between the two parts of China is 9.1, 0.7, −2.5, 0.1, 1.4, 1.3, 2.0, 4.7, and −2.1%, respectively. Furthermore, the attributive differential for the above factors of cerebrovascular disease between the two parts of China is 8.7, 0.0, −5.2, 0.1, 1.0, 0.0, 2.4, 4.7, and −2.1%. Diet high in sodium is the leading cause of the north–south differential in CVD, resulting in 0.71 less years of life expectancy in northern compared with that in southern China.

## Introduction

Cardiovascular disease (CVD), tumors, and respiratory diseases are the three leading causes of death in China in both the rural and urban populations, jointly accounting for ~80% of all-cause mortality (1). The risk factors of diseases can be divided into two categories.

The first category includes geographical and environmental factors, which include temperature, air quality, air pollution, and elevation. Several studies have shown that extreme temperatures have a significant impact on mortality (2, 3). An excess of deaths is observed during both winter and summer (4–7). Typically, a *U*-shaped relationship between temperature and death is observed with mortality risk decreasing from the lowest temperature to an inflection point and then increasing with higher temperatures (8). Studies have also found that extremely cold temperatures can affect deaths occurring not only on that same day but also on several subsequent days, a phenomenon called delayed effects (9). Outdoor and indoor air pollution are leading risk factors for disease burden (10, 11), and PM_2.5_, NOx, SO_2_, and O_3_ can induce CVD and respiratory disease (12–18).

The second factor includes lifestyle issues, which include sodium intake, vegetable and fruit intake, smoking and second-hand smoke exposure, alcohol usage, physical activity, lack of sleep, obesity, and mental status. Physical activity is an effective way to reduce the risk of stroke and heart disease (19). Some studies have demonstrated that moderately intense physical activities will decrease the risk of CVD by 14% [hazard ratio (HR) = 0.86; 95% CI, 0.80–0.93] (20). High dietary sodium intake is the primary dietary risk factor globally, and it is the main cause of hypertension and CVD especially in Eastern Asia and China (21, 22). There is a strong positive correlation between obesity and ischemic stroke, with the risk of ischemic stroke increasing by 30% (HR, 1.30; 95% CI, 1.28–1.33) for every 5 kg/m^2^ increase in body mass index (23). Smoking is associated with 1.3 million cardiovascular events and accounts for approximately one-third of the male CVD burden in China in 2011 (24). Lack of vegetables and fruit is also an important risk factor for disease with the risk of hypertension decreasing with an increase in daily vegetable intake (25).

**Figure 1:**
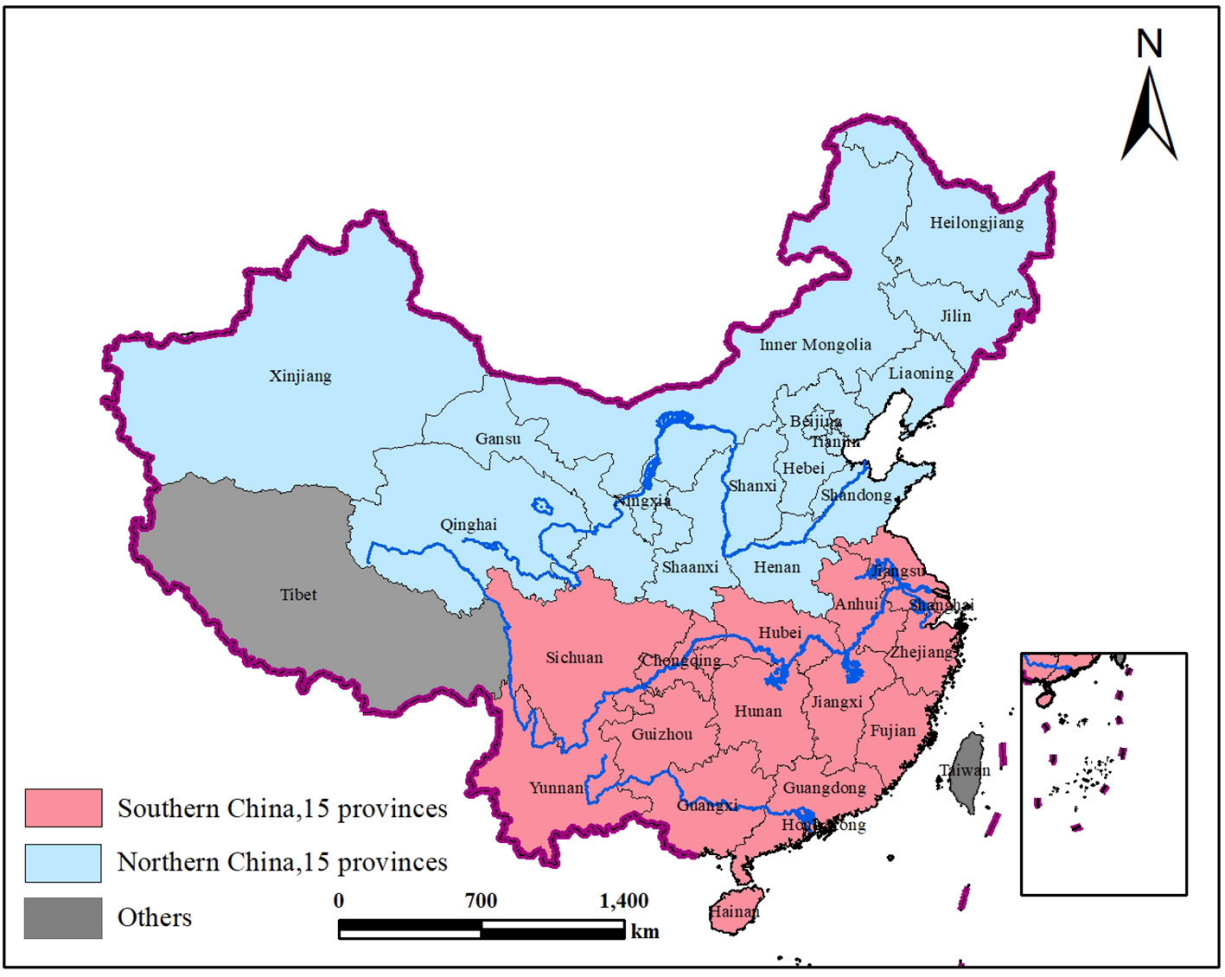
Northern and southern China.

Mainland China has 31 provinces with the north–south boundary of China being Qingling Mountain and the Huai River. This results in southern and northern China each containing 15 provinces, excluding Tibet in either northern or southern China. Although Tibet is geographically located in the south, the average elevation is over 4,500 m, and its temperature, atmospheric pressure, and oxygen content are far lower than those of other provinces, and it is known as the third pole of the Earth. Figure 1 shows the provinces of northern and southern China.

The land of China, as one of the biggest countries in the world, covers a range of 4,000 km from the north to the south, with varying environmental and lifestyle factors. Although the factors noted above have important effects on disease and health, the geographic distribution of each factor and its attribution to the north–south differential within China is obscure. To address these conundrums, this study collected age-standardized mortality rates for all-cause, CVD, tumors, and respiratory diseases data in each province. Statistical tests were used to determine the significant difference of the above diseases between northern and southern China. The leading risk factors for the diseases in each province were collected, and their attributable risk proportion, the standardized mortality rate, and reduced life expectancy were calculated.

## Data and Methodology

### Data

#### Data of Four Major Fatal Diseases

To study the underlying differences in mortality between northern and southern China, the mortality rate of major fatal diseases in China from 2008 to 2017 was reviewed. The data is taken from the yearly China Health Statistics Yearbook edited by the National Health Commission of China (1). Of all deaths in rural and urban areas, CVD-related deaths accounted for 45.50 and 43.16%, respectively, followed by tumor-related deaths, 22.92 and 26.06%, and respiratory disease-related deaths, 12.02 and 11.24%, respectively. Overall, the three diseases accounted for 80.44 and 80.46% of all deaths in rural and urban areas, respectively.

**Figure 2:**
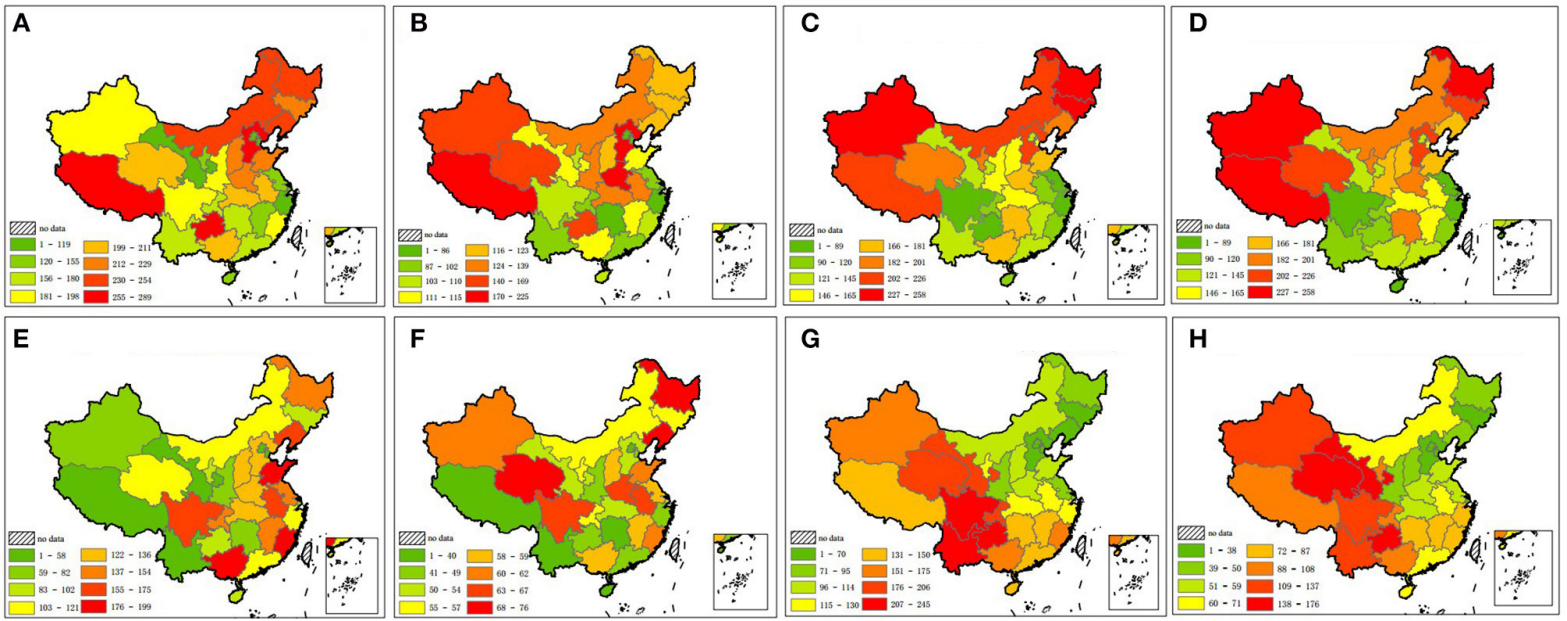
Distribution of age-standardized mortality rates of four major fatal diseases. Warmer colors indicate a higher mortality rate. **(A)** Cerebrovascular disease in male. **(B)** Cerebrovascular disease in female. **(C)** Heart disease in male. **(D)** Heart disease in female. **(E)** Major tumors in male. **(F)** Major tumors in female. **(G)** Respiratory disease in male. **(H)** Respiratory disease in female.

**Figure 3:**
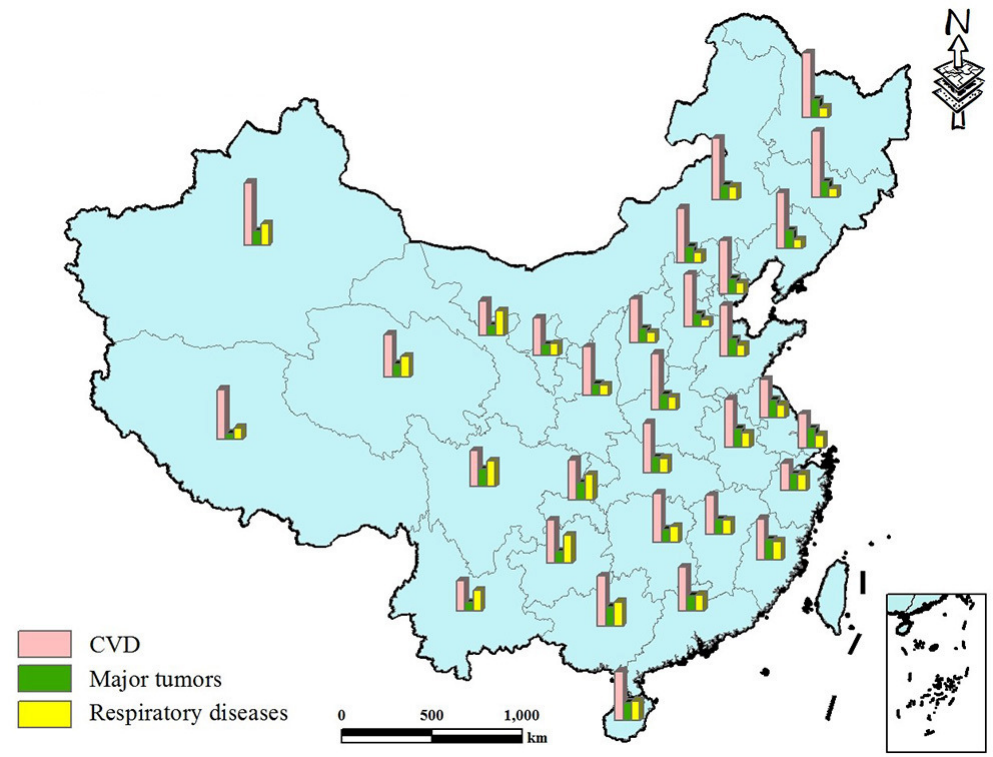
Each disease's percentage of total age-standardized mortality rate.

Cardiovascular disease can be further categorized into cerebrovascular disease and heart disease. Information on the mortality rates of these four major diseases was collected and mapped using provincial mortality rate data (26–28) (Figure 2). In this figure, major tumors include lung, liver, stomach, colon, and rectal cancer. The proportion of each disease and the corresponding total age-standardized mortality rates are summarized in Figure 3.

#### Data Collection and Calculation of Each Risk Factor on CVD

To study why the age-standardized mortality rate for CVD is significantly lower in southern than in northern China, the primary risk factors for CVD in China were reviewed (24). The top lifestyle factors associated with CVD in China include high dietary sodium intake; current smoking; physical inactivity; a diet low in marine omega-3 fatty acids; overweight and obesity; alcohol intake; and a diet low in fruit, fiber, nuts, whole grains, and vegetables. The primary environmental factors are temperature extremes (high and low temperatures) and air pollution (especially PM_2.5_). There are no accurate provincial data on physical inactivity and alcohol intake. Therefore, the following 12 risk factors were studied:

1) Vegetables, nuts, whole grain, fiber, and fruit intake

Data on per-capita vegetable (including vegetables and edible fungi), nuts, whole grain, fiber, and fruit intake from all 31 provinces were obtained from the China Statistical Yearbook (2016, 2017, and 2018, http://www.stats.gov.cn/tjsj/ndsj/) and China Food Composition Tables, Standard Edition (Figures 5A–E).

2) Tobacco

Tobacco exposure is one of the primary risk factors for CVD. Investigation of tobacco exposure involves smoking rate, smoking amount, cessation, relapse rate, etc. Data on smoking rates in the same region collected by different institutions over different periods are quite different. Therefore, data on per-capita tobacco sales, rather than smoking rates, were used. Data on tobacco sales were obtained from statistics on the tobacco sales website in China (http://www.yanb2b.com). The calculation method is shown in Equation 1 with the per-capita tobacco sales data for each province shown in Figure 5F.

(1) $$\begin{array}{l} T_{i}=\frac{K_{i}}{P_{i}},\;P_{i}=L_{i}+M_{i} \end{array}$$

Equation 1: *T*_*i*_, per-capita tobacco sales for each province; *K*_*i*_, tobacco sales for each province; *P*_*i*_, total population of each province; *L*_*i*_, permanent residents of each province; *M*_*i*_, floating population of each province.

3) Sodium intake

Data on regional salt intake varied among different studies. The data for each province in different studies (29, 30) were averaged, and the results are illustrated in Figure 5G.

4) PM_2.5_

**Figure 4:**
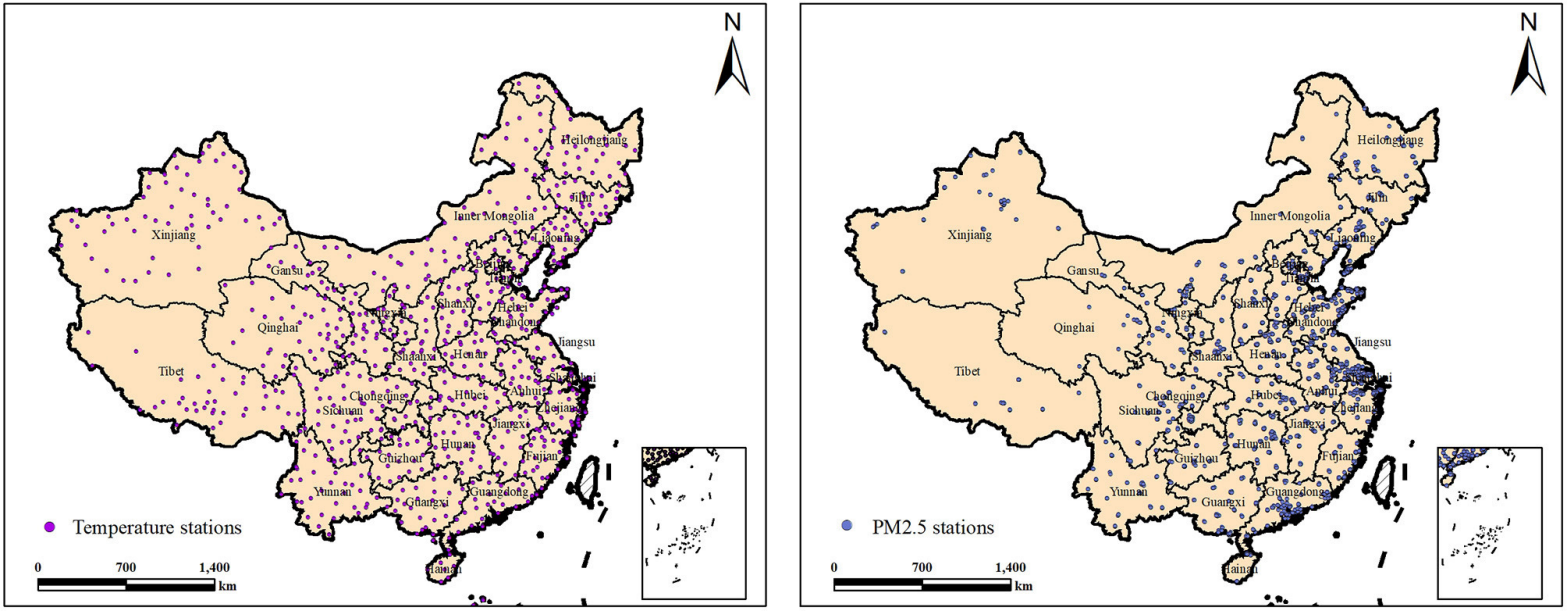
Location of temperature and PM_2.5_ monitoring stations.

Data on PM_2.5_ are from the national urban air quality real-time publishing platform (http://106.37.208.233:20035/) owned by China's Environmental Monitoring Center. The daily PM_2.5_ data from 1,619 stations located in 31 provinces from May 13, 2014, to December 31, 2019, were collected. The distribution of the 1,619 PM_2.5_ monitoring stations is noted in Figure 4 (right). The inverse distance-weighted method was used to produce the PM_2.5_ concentration grid graph of China (resolution, 1 × 1 km). The average PM_2.5_ data of the monitoring stations in each province cannot accurately represent the PM_2.5_ of several residential areas because many provinces comprise both flat and steep mountainous areas with a population density higher in the flat areas. Therefore, a 1 × 1 km population density grid map (http://www.resdc.cn) was collected, and a population density-weighted average PM_2.5_ (PDP) for each province was calculated. Areas with greater populations were given greater weights in the average PM_2.5_ calculation, which can be expressed as follows:

(2) $$PDP_{i}=\frac{\sum_{i=1}^{n}\left(PO_{i}\times PM_{i}\right)}{\sum_{i=1}^{n}PO_{i}}$$

Equation 2: *n*, number of grids for each province; *PO*_*i*_, population of grid *i* (1 km × 1 km); *PM*_*i*_, PM_2.5_ of grid *i* (1 km × 1 km)

**Figure 5:**
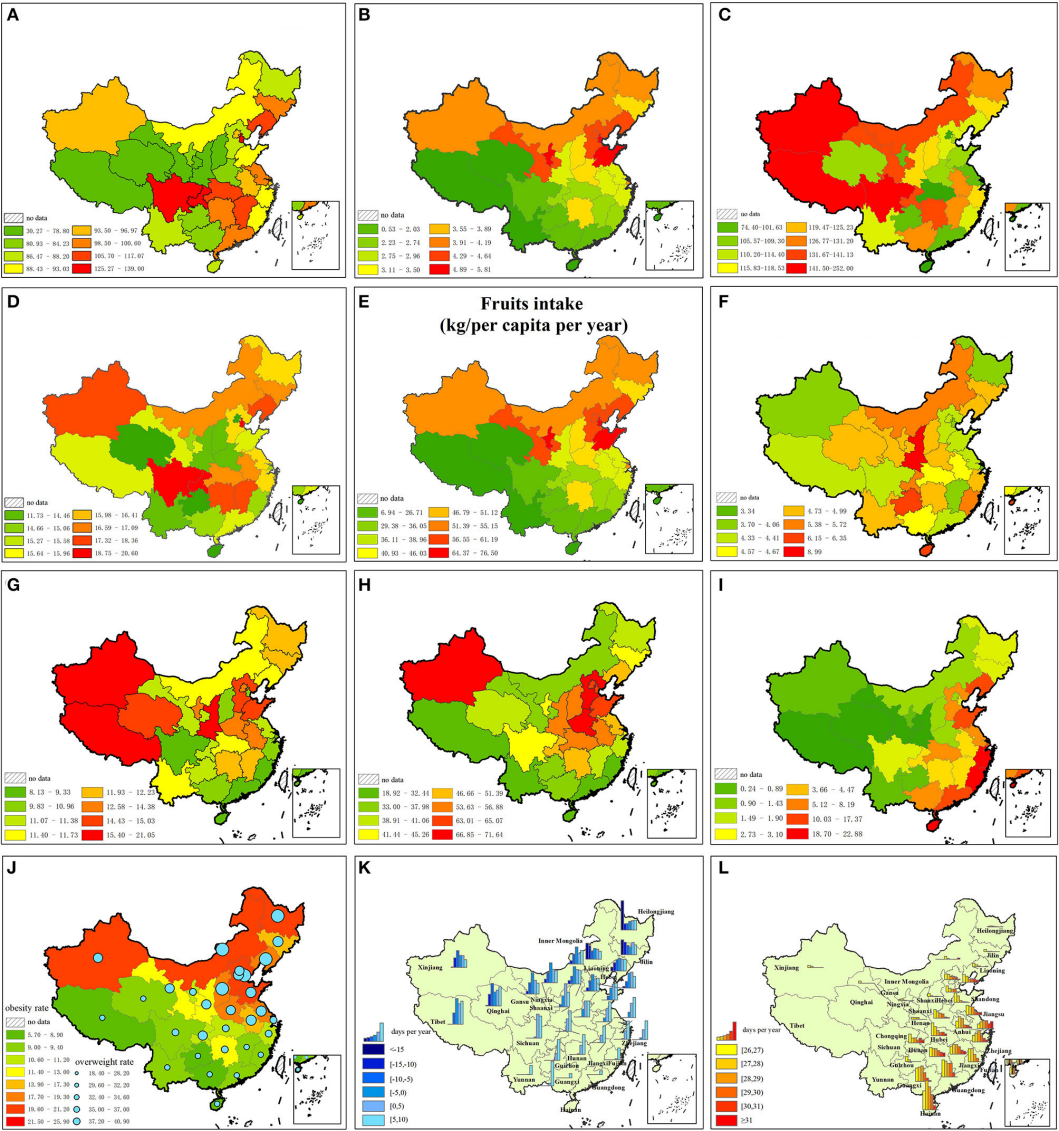
Risk factors for CVD. **(A)** Diet low in vegetables. **(B)** Diet low in nuts and seeds. **(C)** Diet low in whole grains. **(D)** Diet low in fibre. **(E)** Diet low in fruits. **(F)** Smoking. **(G)** Diet high in sodium. **(H)** PM2.5. **(I)** Diet low in seafood omega-3 fatty acids. **(J)** Obesity. **(K)** Low temperature. **(L)** High temperature.

The PDP map was created based on Equation (2), and the results are illustrated in Figure 5H.

5) Seafood intake

Data on seafood intake are from the China Statistical Yearbook (http://www.stats.gov.cn/tjsj/ndsj/) and China Fisheries Statistical Yearbook (2016–2018). The average annual seafood intake of each province from 2016 to 2018 was collected, and the per-capita seafood intake is shown in Figure 5I.

6) Obesity and overweight rate:

Data on obesity and overweight rates are from the Chinese Center for Disease Control and Prevention (31) and represent the data collected in 2013 (Figure 5J).

7) Low and high temperatures

Temperature data are from the China Meteorological Data Network (http://data.cma.cn). Daily temperature data from 756 temperature-monitoring stations across the country in 2000 and 2010 were collected. The geographical distribution of national temperature monitoring stations is shown in Figure 4 (left). By calculating average data for the same day at each provincial station, the daily average temperature for each province was obtained. Relevant studies have shown that an average daily temperature of >26 or <10°C will lead to an increase in death and CVD (32). The risk increases with temperatures of >26 or <10°C. Based on the monitoring data from each station, we calculated the yearly number of days for each temperature range (26–27, 27–28, 28–29, 29–30, 30–31, >31, 5–9, 0–4, −5 to −1, −10 to −6, −15 to −11, <-15) for each province (Figures 5K,L).

### Methods

#### Statistical Methods

The normal distribution and statistically significant difference between the two parts of China for each disease were studied using normal distribution tests (method: Shapiro–Wilk test) and *t*-test. All statistical analyses were performed using SPSS 25, and the statistical significance was set at *p* < 0.05, *p* < 0.01, and *p* < 0.001, respectively.

#### Calculation of Each Disease's Proportion to the North–South Difference in Mortality Rate

To evaluate correlations among the four major diseases and the north–south difference in mortality rate, **Equation 3** was developed.

(3) $$\begin{array}{l} P_{i}=\left(M_{i}^{North}-M_{i}^{South}\right)/\left(M_{t}^{North}-M_{t}^{South}\right) \end{array}$$

*P*_*i*_ indicates the proportion of disease *i* to the north–south division; *M* indicates the age-standardized mortality rate; *t* indicates the total mortality rate of the four diseases; *P*_*i*_ > 0 indicates that the disease is positively related to the north–south division.

#### Attribution of Each Factor to Provincial CVD

To quantitatively assess the attribution of the 12 risk factors on provincial CVD mortality, the estimation of attributable disease burden according to the theory and methodology in the Global Burden of Disease (GBD) was calculated (33). In GBD, the estimation of disease burden attributed to the various risk factors is conducted under the framework of comparative risk assessment theory. The core content of this theory is that when the exposure level of other independent risk factors remains unchanged, the proportion of disease burden attributed to a particular risk factor, the population attribution fraction (PAF), is calculated by comparing the exposure distribution of that factor with the theoretical minimum risk exposure distribution. The formula for PAF is shown in Equation 4, and the attributed death and mortality rate is calculated in Equation 5.

(4) $$PAF=\frac{\sum_{i=1}^{n}P_{i}(RR_{i}-1)}{\sum_{i=1}^{n}P_{i}(RR_{i}-1)+1}$$

*RR*_*i*_, relative risk at exposure level *I*; *P*_*i*_, proportion of population at exposure level *I*; the RR and its 95% CI for each risk factor are from the GBD 2017 (http://ghdx.healthdata.org/gbd-2017).

(5) $$\begin{array}{l} AM=PAF\times M \end{array}$$

*AM*, attributed number of deaths to a risk factor; *M* number of deaths for each CVD.

Calculation of the 12 risk factors can be divided into two categories. First, the attributable risk proportion of PM_2.5_; obesity; smoking; a diet low in vegetable, fruit, omega-3 fatty acids, nuts, fiber, and whole grain content; and a diet high in sodium intake are calculated using Equations 4, 5. The per-capita omega-3 fatty acid intake of each province was calculated based on the provincial seafood intake and average omega-3 fatty content of various seafood products in China.

The second is the determination of the risk of high and low temperatures. Temperature is an important risk factor for CVD. Numerous studies have shown that both a high- and low-temperature environment will increase the incidence of CVD and mortality. Temperature has a lagging effect on CVD. High-temperature effects appear to last for several days, whereas low-temperature effects may persist for up to several weeks (34). The RR of annual high and low temperatures for each province was based on the number of lag days for each high and low temperature (Equation 6).

(6) $$RR_{k}^{one\;year}=1+\sum \left(\frac{\sum_{i=1}^{n}RR_{k}^{i}-n}{365}\times d_{k}\right)$$

*k*, average temperature per day; low temperature *k* ∈ (−25*C*, 9*C*); at high temperature *k* ∈ (26*C*, 33*C*); *n* lag days that RR and its 95% CI are both >1; *RR*_*i*_, relative risk of temperature *k* at day *i* of lag; *i* ∈ (0, 30); *d*_*k*_, number of days in a year that average temperature = *k*.

## Results

### Difference in Mortality Rate Between Northern and Southern China

The provincial data of diseases correspond to normal distribution. Table 1 presents the comparison of differences in mortality rates between northern and southern China.

**Table 1:** Age-standardized mortality rates (1/100,000) and *t*-tests of major diseases between northern and southern China.

	**Index**	**Sample size**	**Position**	**Cerebrovascular disease**	**Heart disease**	**Major tumors**	**Respiratory diseases**	**All causes**
Age-standardized mortality	Average value (STD)	15	North	168.0 (29.6)	170.3 (29.3)	94.1 (14.1)	85.7 (43.0)	573.7 (84.9)
		15	South	143.6 (31.5)	111.0 (30.4)	98.2 (15.5)	117.0 (43.5)	539.9 (78.1)
*t*-tests		*T*-value		2.190	5.437	−0.753	−1.983	0.979
		*P*-value		0.037^*^	0.000^***^	0.458	0.057	0.336

*STD, standard deviation*.

* *Significant at p < 0.05 level*.

**significant at p < 0.01 level.

*** *significant at p < 0.001 level*.

Table 1 shows that cerebrovascular and heart diseases present a statistically significant difference between northern and southern China (cerebrovascular disease, *P* < 0.05; heart disease, *P* < 0.001), with the mortality rate of cerebrovascular and heart diseases higher in northern China than in southern China (northern China: 338.3/100,000; southern China: 254.6/100,000), and the significance of heart disease higher than that of cerebrovascular disease. Although major tumors and respiratory diseases are lower in northern China than in southern China and all-cause mortality rates are higher in northern China than in southern China, there is no obvious north–south difference in the distribution of major tumors (*P* = 0.458), respiratory diseases (*P* = 0.057), and all-cause mortality rates (*P* = 0.336).

Table 2 presents the proportion of major diseases and the corresponding north–south difference in all-cause mortality rate.

**Table 2:** Age-standardized mortality rates with leading causes of death and their proportion on north–south differences in all-cause mortality rate in 2013.

	**Age-standardized mortality rate (1/100,000)**					**Proportion to north–south difference (*P*_*i*_)**			
	**CV**	**HD**	**RD**	**MT**	**Total**	**CV**	**HD**	**RD**	**MT**
Males, north	199.84	196.6	99.18	130.4	626.02	73%	167%	−111%	−29%
Males, south	170.88	129.9	143.4	142	586.18				
Females, north	136.15	144	72.2	57.91	410.26	35%	90%	−32%	7%
Females, south	116.3	92.2	90.68	53.6	352.78				

*CV, cerebrovascular disease; HD, heart disease; RD, respiratory diseases; MT, major tumors*.

In Table 2, *P*_*i*_ (calculated by **Equation 3**) of cerebrovascular and heart diseases of >0 indicates that the diseases are positively related to the north–south division. *P*_*i*_ of heart disease (167% for male and 90% for female), which is higher than that of cerebrovascular disease (73% for male and 35% for female), indicates that the proportion of heart disease is higher than that of cerebrovascular disease. *P*_*i*_ of respiratory diseases of <0 indicates that the disease is negatively related to the north–south division.

### Differences in Mortality Rate Based on 12 Risk Factors of CVD Between Northern and Southern China

**Figure 6:**
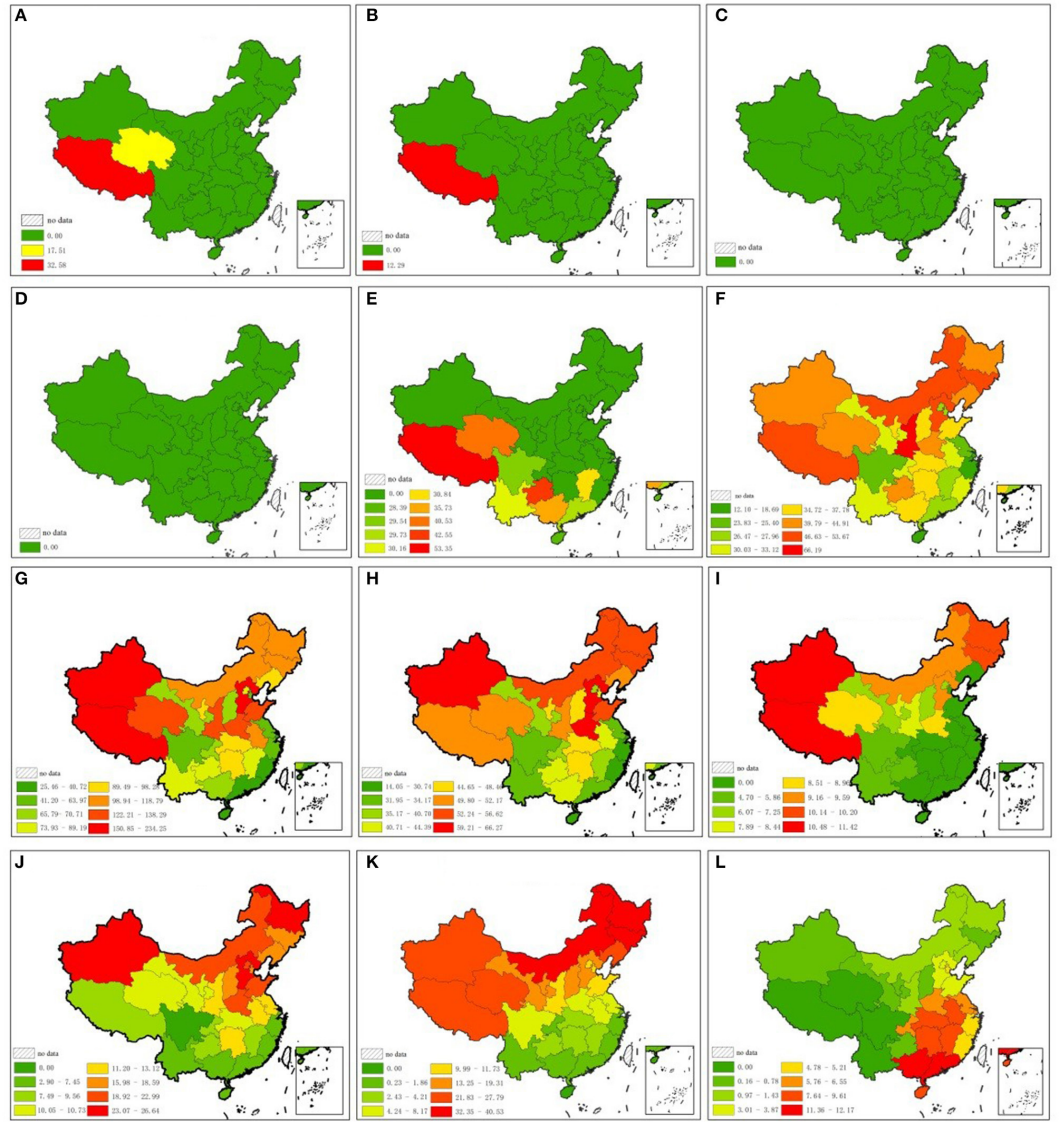
Attributable proportion of 12 risk factors for CVD in each province. **(A)** Vegetables and edible fungi intake (kg/per capita per year). **(B)** Nuts and seeds intake (kg/per capita per year). **(C)** Whole grain intake (kg/per capita per year). **(D)** Fibre intake (kg/per capita per year). **(E)** Fruits intake (kg/per capita per year). **(F)** Tobacco sales (cigarettes/per capita per year). **(G)** Salt intake (g/per capita per year). **(H)** PM_2.5_ (μg/m^3^). **(I)** Seafood intake (kg/per capita per year). **(J)** Obesity and overweight rate. **(K)** Yearly number of days for each low temperature range. **(L)** Yearly number of days for each high temperature range.

The standardized CVD mortality rate of the 12 risk factors for each province is illustrated in Figure 6. The average value of attributable risk proportion in 15 northern and 15 southern provinces is illustrated in Tables 3, 4. The reduction in life expectancy caused by each risk factor for CVD is illustrated in Table 5.

**Table 3:** Attributable fraction of risk factors for heart disease in northern and southern China.

	**Sodium**	**Vegetable**	**Fruit**	**Smoking**	**PM_**2.5**_**	**Omega-3**	**Obesity**	**LT**	**HT**
Northern China (% and 95% CI)	38.1%	0.7%	0.5%	14.7%	18.1%	4.3%	4.7%	5.6%	0.6%
	21.0–70.5%	0.3–1.0%	0.2–0.7%	11.6–20.8%	16.1–20.1%	1.8–7.0%	4.0–7.4%	3.0–8.1%	0.5–0.8%
Southern China (% and 95% CI)	29.0%	0%	3.0%	14.6%	16.7%	3.0%	2.7%	0.9%	2.7%
	15.0–65.8%	0.0–0.0%	1.1–4.5%	11.5–20.6%	14.6–18.7%	1.4–5.4%	2.3–4.4%	0.5–1.3%	2.0–3.3%
Differential	9.1%	0.7%	−2.5%	0.1%	1.4%	1.3%	2.0%	4.7%	−2.1%

*LT, low temperature; HT, high temperature*.

**Table 4:** Attributable fraction of risk factors for cerebrovascular disease in northern and southern China.

	**Sodium**	**Vegetable**	**Fruit**	**Smoking**	**PM_**2.5**_**	**Omega-3**	**Obesity**	**LT**	**HT**
Northern China (% and 95%CI)	34.9%	0.0%	1.0%	9.5%	12.3%	0.0%	5.9%	5.6%	0.6%
	24.0–44.4%	0.0–0.0%	0.6–1.5%	8.9–22.8%	9.6–14.3%	0.0–0.0%	4.2–9.5%	3.1–8.0%	0.5–0.8%
Southern China (% and 95%CI)	26.2%	0.0%	6.2%	9.4%	11.3%	0.0%	3.5%	0.9%	2.7%
	17.4–34.6%	0.0–0.0%	3.5–9.1%	8.7–22.6%	8.9–13.3%	0.0–0.0%	2.5–5.7%	0.5–1.3%	2.0–3.3%
Differential	8.7%	0.0%	−5.2%	0.1%	1.0%	0.0%	2.4%	4.7%	−2.1%

**Table 5:** Attributable fraction of risk factors for reduced life expectancy caused by CVD (year).

**CVD**	**PM_**2.5**_**	**Sodium**	**LT**	**HT**	**Fruit**	**Vegetable**	**Obesity**	**Smoking**	**Omega-3**
Northern China	0.553	1.398	0.118	0.012	0.023	0.017	0.201	0.377	0.078
Southern China	0.384	0.688	0.012	0.038	0.121	0.000	0.078	0.253	0.042

Residents in northern China consume more sodium (2.32 g/per capita per day higher than southern China), more fruits (16.07 kg/ per capita per year higher than southern China), fewer vegetables (12.04 kg/ per capita per year lower than in southern China), and less seafood products (5.86 kg/ per capita per year lower than in southern China), and tend to be overweight (7.52% higher than in southern China). PM_2.5_ is higher in northern China than in southern China (13.16 μg/m^3^ higher than in southern China). Diet low in nuts and seeds, whole grains, and fiber is not related to CVD in China because the provincial intake is higher than the minimum safety value (Figure 6, Tables 3–5). Attributable risk proportion of sodium, vegetable, PM_2.5_, omega-3, obesity, and low temperature is higher in northern China than in southern China, whereas that of fruit and high temperature is lower in northern China than in southern China. Smoking is approximately balanced between northern and southern China; the differential of attributable fraction is 0.1 for both heart disease and cerebrovascular disease. Sodium is the leading cause of north–south differential for CVD; the differential of attributable fraction is 9.1% for heart disease and 8.7% for cerebrovascular disease. The total attributive differential for the 12 risk factors for heart disease (14.7%) between northern and southern China is higher than that of cerebrovascular disease (9.6%), which is in accordance with the result of *t*-tests.

## Discussion

The distribution of cerebrovascular disease (*P* = 0.037) and heart disease (*P* = 0.000) was positively related to the north–south differential for age-standardized mortality rates because mortality owing to cerebrovascular and heart diseases is higher in northern China than in southern China. Except for cerebrovascular and heart diseases, the sum of all other causes for mortality was higher in southern China (285.3/100,000) than in northern China (235.4/100,000). Therefore, the difference in CVD mortality rate is the fundamental reason for the difference in mortality rate between northern and southern China.

The mortality rate from CVD is generally lower in southern China than in northern China, which is consistent with the findings of other studies. Gelin et al. (35) found that nine provinces in northern China, Heilongjiang, Jilin, Liaoning, Inner Mongolia, Hebei, Beijing, Ningxia, Tibet, and Xinjiang, have a high incidence of stroke, essentially constituting a stroke belt. The stroke incidence in this zone was 236.2/100,000, which was significantly higher than that in areas outside this zone (109.7/100,000). Similar findings have been noted in other countries where a stagnating decline in CVD mortality was the primary cause of no increase in life expectancy since 2010 (36).

Among the 12 risk factors, exposure to six of the factors is higher in northern China than in southern China. Residents living in northern China have a lower vegetable intake, higher sodium intake, higher PM_2.5_ exposure, lower intake of seafood products, higher obesity and overweight rate, and exposure to lower temperatures than those living in southern China. However, those living in southern China have a higher exposure risk to only lower fruit intake and exposure to higher temperatures. There was no obvious difference between northern and southern China in the exposure risk to tobacco, whole grain, fiber, and nuts.

Salt intake is significantly higher in northern China than in southern China. A diet high in sodium has the greatest impact on blood pressure. A total of 40% of hypertensive incidents are caused by a high salt-containing diet, and hypertension is the primary risk factor for CVD and all-cause mortality in the GBD (33). Sodium exposure is a leading risk factor for CVD in China, which is similar to the results of other studies (21, 22), and sodium exposure is also the leading cause of the north–south differential in CVD mortality rate.

Regions with generally higher PM_2.5_ concentrations are located in northern China, particularly in areas around Beijing. It is the area with the most intensive heavy industry and consumes the greatest quantity of coal. Additionally, China's Huai River Policy (Huai River is 0 centigrade isotherm in January in China) proposed in the 1950's provides free or heavily subsidized coal for indoor heating during winter to regions north of the Huai River but not to those to the south; the rural residents usually use solid fuel for heating. This results in a large amount of particulate matter emission in northern China; it causes more death and reduces life expectancy (37, 38). In the current study, PM_2.5_ is the primary reason for the north–south differential in CVD mortality rate.

The rates of obesity and overweight are significantly higher in northern China than in southern China. The 10 provinces with the highest obesity rates are all located in the north. The result is consistent with those of other studies. Northern residents, including non-Han ethnic groups, have significantly taller and larger body mass than southern residents (39). Studies found that there are six gene frequencies that are different among people from both northern and southern China. A variation in the gene FADS2 is more commonly found in the northern population than in the southern population. This gene helps people metabolize fatty acids, which suggests a diet rich in high animal fat content, affects the dietary differences between northern and southern China, and affects the body shape (40, 41).

Seafood intake is higher in the south than in the north. This is primarily owing to the geographic differences between the two regions. Among the 11 coastal provinces in China, seven are located in the south and only four in the north. Southern China has a longer coastline, vaster and deeper sea area, more fishing grounds, and more abundant fishery resources. This results in the daily consumption of seafood being higher in southern China than in northern China. This study found that there are 23 provinces, Tibet, 13 northern provinces, and 9 southern provinces, with <100 mg/day omega-3 fatty acids intake (the 2017 GBD standard).

Although both intensely hot and cold temperatures will cause an increase in the mortality rate (42), the relationship between the two extremes and death is different. Some studies have reported more cold-related than heat-related deaths (43–45). The effect of cold temperature persists for several days (46, 47), whereas that of high temperature is limited to the day of death or the immediately preceding day (48). Additionally, winter temperature is much lower in northern China than in regions with the same latitude in the world, and the temperature in January in northeastern China is usually as low as −15-−25°C, which is far lower than that in southern China, whereas differential summer temperature between the two parts of China is far less than the winter temperature. This explains the higher number of deaths caused by extreme temperature in northern China than in southern China.

To the best of our knowledge, this was the first study examining the relationship between dietary and environmental factors and death between southern and northern China. These results provide evidence for differences in CVD caused by the geographical environment and lifestyle. The winter in northern China is colder than regions of the same latitude throughout the world. The temperature in January in northeastern and northwestern China is lower than −20°C, although the latitude of most residential areas is <50° N, whereas it is >20°C in Hainan Island in southern China. These large temperature differences contribute to significantly different lifestyles and eating habits.

There are some limitations to this study. First, because it was difficult to collect all data, not all risk factors affecting mortality were included; i.e., physical inactivity and alcohol intake are important risk factors of CVD, but we did not find accurate data on provincial physical inactivity. Because many types of wine are not only produced by wineries but also brewed by residents themselves, it is very difficult to obtain provincial alcohol consumption data in China. In addition, the alcohol content of different types of wine varies greatly. Hence, only 12 risk factors for CVD were analyzed on a provincial scale. Second, GBD estimates aggregated studies across the world, but these do not necessarily apply to the Chinese population as the risk factor and disease patterns in China may be substantially different from the “global average.” Errors are inevitable in estimating the relative risk of China using GBD data. Third, the periods of risk factors are not entirely consistent with each other, which affects the accuracy of the results because the dose of each factor varies over the years. For example, PM2.5 in China decreases year by year, and the 2014–2019 PM2.5 data may exaggerate its attributable risk compared with the 2016–2018 data on diet factors.

## Conclusion

This study examined the north–south difference in the mortality rate of major diseases. The proportion of heart disease, cerebrovascular disease, major tumors, and respiratory disease on the differential for all-cause mortality rate in northern and southern China was calculated. The results found that the mortality rate of cerebrovascular and heart diseases is statistically higher in northern China than in southern China.

A total of 12 important risk factors for CVD were selected to try to determine reasons for the regional difference in mortality. Based on the relative risk for each factor in GBD 2017, the PAF of each of the 12 factors in each province was calculated, and the age-standardized CVD mortality rate attributed to each factor was obtained.

The results found that the residents of northern China have higher exposure and attributable risk proportion to six risk factors. They eat more sodium, fewer vegetables, and less sea products, and they are likely to be overweight. The PM_2.5_ is higher in northern China than in southern China. Lastly, cold temperatures cause a greater number of deaths than hot temperatures owing to the fact that northern China is colder than regions in the world with the same latitude. All these factors lead to a higher CVD mortality rate in northern China.

## Data Availability Statement

Publicly available datasets were analyzed in this study. This data can be found at: http://106.37.208.233:20035, http://www.stats.gov.cn/tjsj/ndsj/, http://data.cma.cn.

## Author Contributions

MW, YS, JC, and XL collected the data and drew the maps. YH designed ideas of the paper and was a major contributor in writing the manuscript. All authors read and approved the final manuscript.

## Conflict of Interest

The authors declare that the research was conducted in the absence of any commercial or financial relationships that could be construed as a potential conflict of interest.
